# Disseminated infections due to Immune Reconstitution Inflammatory Syndrome after Highly Active Antiretroviral Therapy-Report of 3 cases from Nigeria

**DOI:** 10.4314/pamj.v9i1.71216

**Published:** 2011-08-16

**Authors:** Dimie Ogoina, Victor Adekunle, Reginald Obiako, Abdulaziz Umar, Michael Akolawole, Joseph Ovosi

**Affiliations:** 1 Department of Medicine, Bingham University Teaching Hospital, Jos, Plateau state, Nigeria; 2 Department of Medicine, Ahmadu Bello University Teaching Hospital (ABUTH), Zaria, Nigeria

**Keywords:** HIV, HAART, Immune Reconstitution Inflammatory Syndromes, IRIS tuberculosis, VZV, HERPES, Nigeria

## Abstract

Immune Reconstitution Inflammatory Syndromes (IRIS) are exaggerated pathological inflammatory reactions occurring after initiation of highly active antiretroviral therapy (HAART) due to exuberant immune responses to occult or apparent opportunistic infections or cancers. In view of paucity of studies from Nigeria, we report 3 cases of IRIS presenting as disseminated infections in HIV-1 infected patients initiating HAART. The first case was a previously healthy female who developed disseminated tuberculosis after 4 weeks of regular HAART. Her HAART regimen was continued and she improved after commencement of anti-tuberculosis drugs, with evidence of progressive increase in CD4 cell count. The second case was a HAART-experienced female who stopped her drugs for 4months. Two months after recommencement of her previous HAART regimen, she developed features of disseminated herpes zoster infection, despite evidence of decrease in viral load by 95%. HAART was continued and she recovered completely after receiving valaciclovir tablets and antibiotics. The third patient was a female student who was commenced HAART on account of chronic cough and weight loss. Three months after regular HAART, she developed features of disseminated Kaposi's sarcoma involving the skin, oropharynx and lungs, despite evidence of 42% increase in CD4 cell count. Unfortunately, she rapidly deteriorated and died during the course of management. Clinicians should be alert to the possibility of IRIS in HIV-infected patients initiated or re-initiated on HAART. There is need for future prospective studies determining risk factors for IRIS in HIV-infected patients from Nigeria.

## Introduction

Highly active antiretroviral therapy (HAART) suppresses HIV-replication and restores immune function ultimately improving the quality of life and prolonging survival of HIV-infected patients. Unfortunately, during HAART-induced restoration of immune function, some patients may experience an exaggerated pathological inflammatory response termed immune reconstitution inflammatory syndrome (IRIS)[1]. IRIS has been reported in 10-40% of patients initiating HAART [2, 3]. Although IRIS may not significantly portend poor long term prognosis [4], it increases the rate of hospitalisation thereby increasing the cost of care of affected individuals [2].

In view of prevailing high HIV prevalence and high rate of new infections, as well as increasing access to HAART, many HIV-infected individuals in sub-Saharan Africa are at a risk of developing IRIS on initiating HAART. However, there is still paucity of studies on IRIS from countries such as Nigeria where an estimated 4500 new HIV infections occur yearly and more than 500 000 HIV-infected patients are expected to initiate HAART [5]. For the first time from Nigeria, we report 3 peculiar cases of disseminated infections due to IRIS in HIV-1 infected patients receiving HAART at Ahmadu Bello University Teaching Hospital, Zaria, Kaduna State.

## Case report

A 37-year old housewife was commenced HAART-(Emtricitabine/Tenofovir/Nevirapine) after 1year progressive weight loss and recurrent diarrhoea, without fever or cough or radiological features of pulmonary tuberculosis. Four weeks after regular HAART, she presented with non-productive cough, breathlessness, fever and progressive painful bilateral inflammatory cervical adenopathies. Chest examination was normal. A repeat chest X-ray however, revealed features suggestive of perihilar adenopathies. Histology of biopsied cervical node confirmed tuberculosis-(TB) granuloma. A diagnosis of disseminated TB-(lungs and nodes) secondary to IRIS was made. A full blood count revealed a packed cell volume of 28% and total white cell count of 2.9 X 10^3^/l, with predominant neutrophils (52%) and lymphocytes (40%). Renal and liver function tests were normal. Standard combination anti-TB drugs by DOTS were commenced along with tablets prednisolone 40mg daily (tapered). Her HAART regimen was continued but Nevirapine was switched for Efavirenz to avoid drug interactions. All symptoms gradually resolved and by the 3rd month on HAART, her CD4 cell count had increased from 15cells/ul to 163cells/ul.

### Case 2

**Figure 1:**
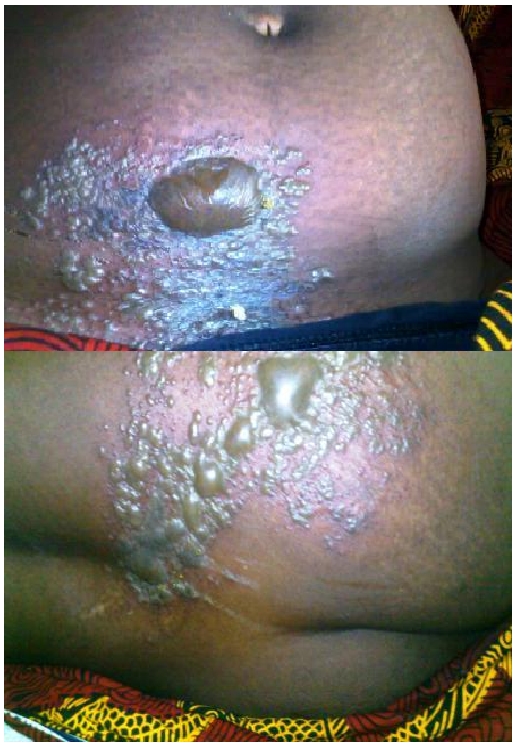
Multidermatomal herpes zoster in a female with Disseminated infections due to Immune Reconstitution Inflammatory Syndrome after Highly Active Antiretroviral Therapy

A 47-years old previously healthy HIV-1 infected female was recommenced HAART-(Emtricitabine/Tenofovir/Nevirapine) after four months of non-compliance. Two months after re-initiation of regular HAART, she developed high grade fever, cough and painful vesicobullous eruptions extending from the abdomen to the buttocks within the T11- L2 dermatomes (Figure 1). Cough was productive of mucoid sputum with associated breathlessness at rest and scattered coarse crepitations in both lung fields. On HAART, her HIV viral load had dropped from 27873 copies/ml to 1267 copies/ml but the CD4 cell count changed slightly (266 to 267cells/ul). Full blood count revealed a packed cell volume of 30% and total white cell count of 4.8 X 10^3^/L, with predominant neutrophils (58%) and lymphocytes (35%). Renal function tests were normal. Chest-X-ray revealed bilateral interstitial opacities. Sputum analysis for bacteria including mycobacteria was negative A clinical diagnosis of disseminated herpes zoster infection (skin and lungs) secondary to IRIS was made. Her previous HAART regimen was continued and tablets valaciclovir 1g bd, intravenous ceftriazone 1g daily and analgesics-Diclofenac potassium 50mg bd were commenced, along with dressing of ulcers. She recovered and continues to do well on follow up.

### Case 3

**Figure 2:**
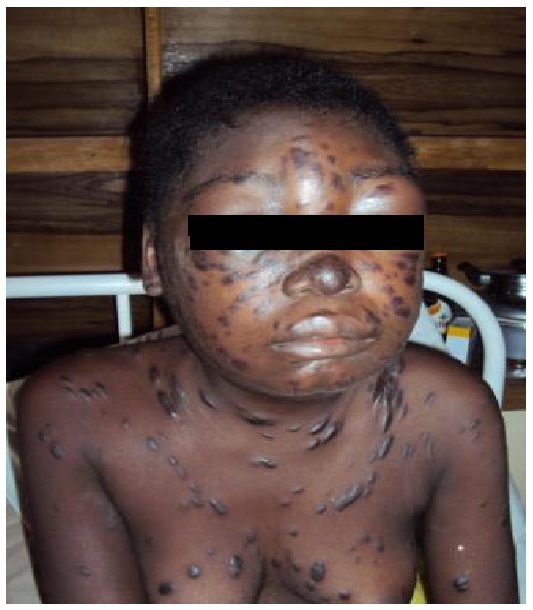
Disseminated Kaposi's sarcoma due to Immune Reconstitution Inflammatory Syndromes (IRIS)-multiple papulonodular dark-coloured generalised skin lesions with oedema especially involving the face

A 19-years old HIV-1 infected female student was commenced HAART-(Tenofovir/Emtricitabine/Efavirenz) and empirical anti-TB drugs on account of eight months history of recurrent fever and weight loss, and one month history of cough with haemopytsis. After three months on regular HAART and three weeks on anti-TB drugs, she developed papulonodular discoloured skin lesions associated with oedema (Figure 2), painful generalised lymphadenopathies and worsening cough and breathlessness. On chest examination she was severely breathless with wide spread coarse crepitations in both lung fields. Other systemic examinations were not remarkable. While on HAART her CD4 cell count had increased from 172cells/ul to 297cells/ul. At presentation, her packed cell volume was 32% and total white cell count was 4.5 X 10^3^/L, with predominant neutrophils (63%) and lymphocytes (26.4%). Sputum microscopy and cytology were negative for AFB and malignant cells respectively. Chest X-ray showed diffuse bilateral alveolar opacities with obliteration of cardiac borders. Skin biopsy confirmed Kaposi's sarcoma but histology of nodal fine needle aspirates revealed non-specific chronic inflammation. A diagnosis of disseminated Kaposi's sarcoma (Skin, nodes, lungs) secondary to IRIS was made. Her previous HAART regimen was continued and anti-tuberculosis drugs were stopped since symptoms did not improve after 2 months of therapy. While being worked up for systemic chemotherapy, she was received tablets prednisolone 60mg daily (tapered), and tablets levofloxacin 500mg daily for empirical coverage of bacterial pneumonia. After an initial improvement, her condition rapidly deteriorated and she died shortly thereafter. Unfortunately, permission for post-mortem was denied on cultural grounds.

## Discussion

The clinical presentations of our patients are consistent with established case definitions for IRIS [6, 7]. IRIS is an inflammatory disease, the consequence of exaggerated dysregulated immune-antigen interactions following HAART-induced immune restoration [1]. During IRIS, occult infections may unmask (e.g. case 1 and 2) or pre-existing infections may exacerbate paradoxically, as exemplified in the case with Kaposi's sarcoma, a human herpes virus 8-induced malignancy known to resolve with HAART [8].

Profound immunosuppression as reflected in low baseline CD4 cell count was a risk factor for IRIS [1] identified in our patients. The disseminated clinical presentation may probably be due to high antigen burden prior to HAART initiation [1], with IRIS promoted by an exuberant immune reconstitution [1] evident by excellent immunological and virological response (e.g. viral load dropped by 95% in case 2) to HAART.

To diminish the exuberant inflammatory response and improve recovery, anti-inflammatory drugs such as steroids remain the cornerstone in the management of moderate to severe IRIS [9]. Although post-mortem diagnosis was impossible, it is our view that progressive lung Kaposi's sarcoma, which was initially misdiagnosed as pulmonary tuberculosis, led to the death of our patient. This case emphasizes the need for clinicians to be vigilant to the possibility of pulmonary KS in HIV-infected patients presenting with cough and haemopytsis. Unfortunately, pulmonary KS is notorious for high fatality, even with systemic chemotherapy [10, 11].

## Conclusion

We have described three cases of disseminated infections following HAART-induced immune reconstitution in HIV-1 infected adult Nigerians. It is desirable for future studies to investigate the clinical spectrum and risk factors of IRIS in adult Nigerians.
